# Duodenal Polyposis: An Incidental Finding of Duodenal-Type Follicular Lymphoma

**DOI:** 10.7759/cureus.73237

**Published:** 2024-11-07

**Authors:** Rachael Hagen, Jasmine Tidwell, Emily Weng, Steven A Goldenberg, Anjiya Shaikh

**Affiliations:** 1 Internal Medicine, University of Connecticut, Farmington, USA; 2 Internal Medicine, University of Connecticut Health, Farmington, USA; 3 Gastroenterology and Hepatology, University of Connecticut Health, Farmington, USA; 4 Gastroenterology and Hepatology, Yale New Haven Hospital, New Haven, USA

**Keywords:** duodenal cancer, duodenal follicular lymphoma, duodenal polyps, duodenal-type follicular lymphoma, radiotherapy (rt)

## Abstract

Duodenal-type follicular lymphoma is a newly recognized and rare variant of follicular lymphoma with a good prognosis. Patients may present with non-specific gastrointestinal symptoms, but they are often asymptomatic. Diagnosis usually occurs incidentally during EGD when duodenal polyps are biopsied. We describe the unique case of incidentally found biopsy-proven duodenal-type B cell follicular lymphoma. Secondary involvement by systemic type B-cell lymphoma was excluded. The patient underwent localized radiotherapy, obtaining complete remission six months later, confirmed by pathology. Physicians should be aware of this rare pathology, considering the potential for extra-duodenal systemic B-cell lymphoma.

## Introduction

Duodenal polyps commonly present as incidental findings on esophagogastroduodenoscopy (EGD). These are often non-neoplastic and represent regenerative or hyperplastic nodules [1,2]. Most neoplastic lesions (11%) are either adenomatous of intestinal or gastric phenotype [2]. In 2017, a new entity of duodenal polyposis was recognized: duodenal-type follicular lymphoma (D-FL) [3]. D-FL is a specific variant of follicular lymphoma, a subtype of low-grade non-Hodgkin lymphoma characterized by a generally benign evolution [3]. Approximately 4% of primary gastrointestinal lymphomas are D-FL [4]. We present a rare case of biopsy-proven low-grade D-FL, found incidentally on a screening EGD in the setting of duodenal polyposis.

This case report was previously presented at the 2022 American College of Gastroenterology Annual Conference in Charlotte, North Carolina, held on October 21-26, 2022.

## Case presentation

A 58-year-old non-smoking male, with a past medical history of hyperlipidemia, presented to an outpatient gastroenterology clinic due to chronic gastroesophageal reflux disease (GERD) refractory to proton pump inhibitor therapy. He had never undergone any endoscopic procedures. His family history was notable for gastric carcinoma in his father, diagnosed at 58 years old.

**Figure 1:**
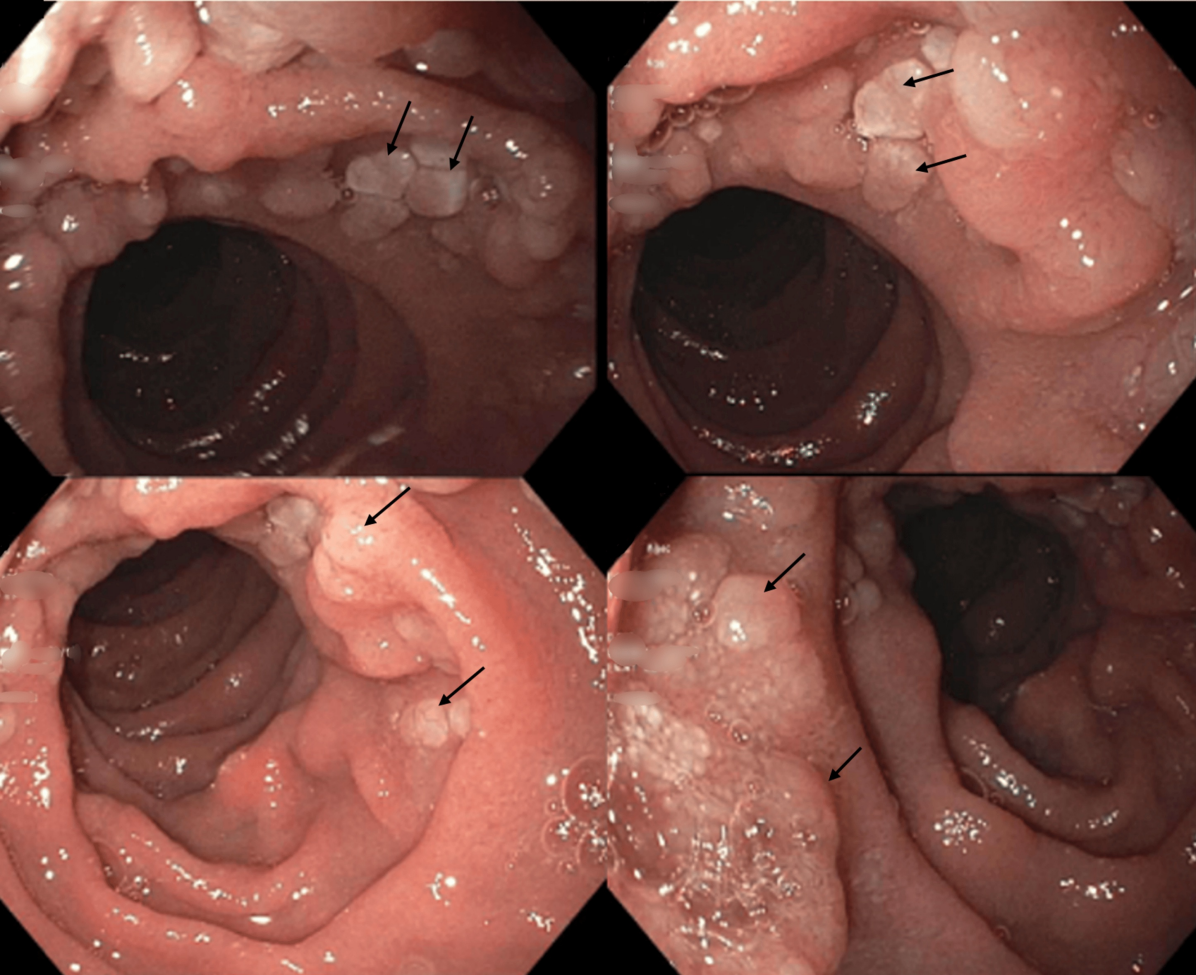
EGD showing duodenal polyposis with multiple semi-sessile polyps (black arrows) throughout the second portion of the duodenum, consistent with duodenal-type follicular lymphoma.

**Figure 2:**
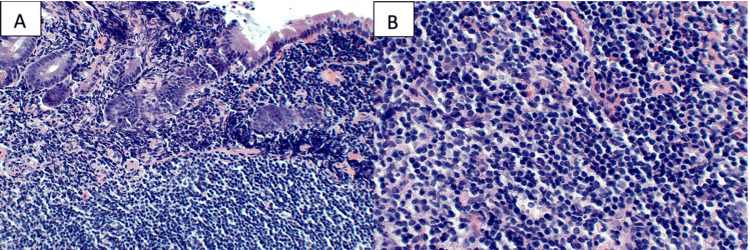
The duodenal histopathology showed prominent lymphoid aggregates, comprising mainly CD20+ B-cells. B-cell follicles were positive for BCL2, BCL6, and CD10 with a low Ki-67 proliferation index. A (20x) and B (40x)

The patient underwent an EGD due to his family history of gastric carcinoma and concern for *Helicobacter pylori*, given symptomatic GERD refractory to treatment and a history of living in Brazil, an endemic region. The EGD revealed an irregular Z-line and patchy inflammation in the gastric body and antrum. Duodenal polyposis was noted with multiple semi-sessile polyps in the second portion of the duodenum (Figure 1). Pathology obtained via cold forceps biopsies revealed moderate acute and chronic gastritis, with a positive immunohistochemical (IHC) stain for *H. pylori*. The duodenal mucosa had abundant lymphoid aggregates, comprising mainly CD20+ B-cells. B-cell follicles were positive for BCL2, BCL6, and CD10 (Figure 2). In conjunction with a markedly nodular duodenum, these findings raised suspicion for D-FL.

The patient was referred to hematology/oncology for further management. Laboratory studies were largely unremarkable, with a normal complete blood count and borderline high uric acid at 8.0 mg/dL and lactate dehydrogenase (LDH) at 226 u/L. Unfortunately, interleukin 2 levels were not obtained. An unremarkable bone marrow biopsy and a whole-body PET scan did not show evidence of fluorodeoxyglucose (FDG) avid malignancy, excluding secondary involvement by a systemic B-cell lymphoma. The patient was diagnosed with a grade 2, stage-one D-FL according to the Lugano classification system.

**Figure 3:**
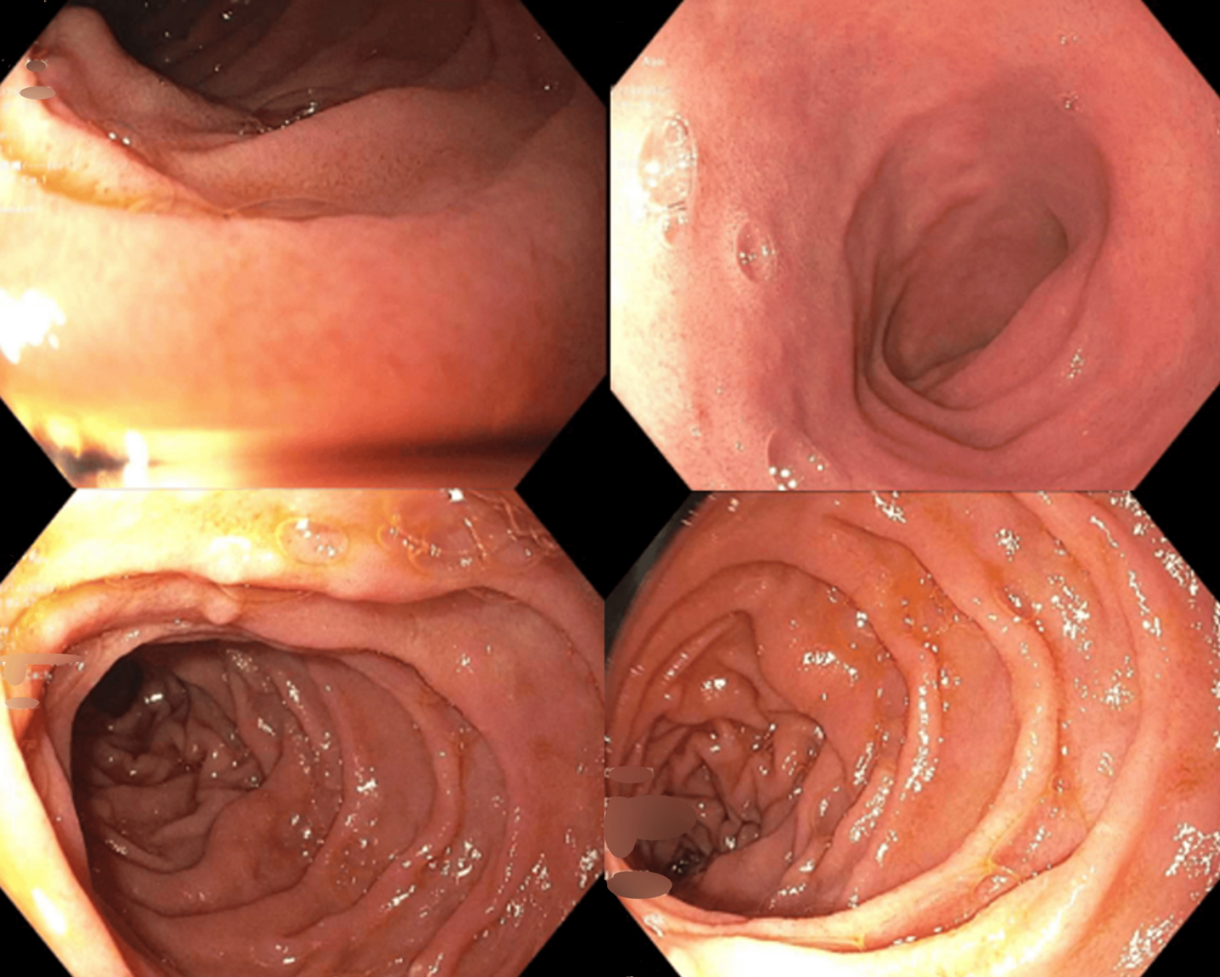
EGD showing a normal appearing duodenum six months following localized radiation therapy.

The patient’s chronic GERD symptoms were attributed to *H. pylori* gastritis, which was treated with quadruple therapy with improvement in symptoms. He was then referred to radiation oncology to discuss treatment options, including localized radiotherapy, involved-field radiotherapy with a curative intent (IFRT), or expectant monitoring off therapy. The patient opted for and underwent IFRT for two and a half weeks. Surveillance EGD performed six months post-radiation therapy showed significant improvement, revealing a normal duodenum (Figure 3). The obtained duodenal biopsies were negative for D-FL, suggesting endoscopic remission. He was followed up with oncology clinic visits every six months, with plans for a repeat EGD if signs of recurrence arose.

## Discussion

D-FL is a newly recognized variant of follicular lymphoma with distinctive clinical and biological features. Patients can present with nonspecific upper gastrointestinal symptoms, such as dyspepsia, fever, weight loss, or anemia [1]. However, most patients are asymptomatic, for which D-FL is usually found incidentally on routine EGDs [1,4,5]. The most common endoscopic finding is the presence of white nodular lesions confined within the second part of the duodenum, consistent with the appearance and location of our patient's lesion [1,3]. Occasionally, lesions appear in other areas of the small intestine [1,3,5]. Lesions are typically low-grade, and IHC staining is often positive for CD20 and BCL2 [3].

There is minimal data regarding treatment options for D-FL since it is a novel pathology only recognized within the last decade [3]. The National Comprehensive Cancer Center guidelines recommend initial treatment of follicular lymphoma with an anti-CD20 antibody-based chemoimmunotherapy, but guidelines pertaining specifically to D-FL are lacking [6]. Staging of D-FL with PET/CT dictates treatment options: most tumors are low-grade with a low clinical stage [7]. Four primary therapeutic options have been studied: watchful waiting, radiation therapy, immunotherapy with rituximab, and immunochemotherapy [3].

D-FL tends to have an excellent prognosis and rare progression, even in the absence of treatment [8]. Low-grade D-FL responds well to IFRT and should be considered with curative intent. Schmatz et al. found IFRT to be curative in 100% of patients in a small case series of 19 patients [9]. If D-FL has progressed further than stage one, expectant management with symptomatic treatment is typically performed, and watchful waiting with annual EGD follow-up is often preferred by oncologists [8]. Kam et al. found an 86.3% five-year progression-free survival with watchful waiting in their study of 23 patients diagnosed with D-FL [8]. Similarly, Matysiak-Budnik et al. reported that 70% of patients experienced spontaneous remission in their study. However, 9% of the patients had transformation into a high-grade lymphoma [5]. There are no defined surveillance recommendations. Serum IL-2 receptor levels are emerging as a marker for monitoring relapse or progression of D-FL [10].

Given that some patients have had histologic transformation into diffuse large B-cell lymphoma [4], individualized treatment options and regular follow-up are important [11]. Immunotherapy with rituximab monotherapy, a CD-20 antagonist, is typically only used in higher stages of D-FL with reported success [4]. In a few cases where resistance to rituximab was described, treatment was ultimately achieved with the addition of bendamustine [12]. Other immunochemotherapies that have proven effective include rituximab combined with chemotherapy agents, such as cyclophosphamide, doxorubicin, vincristine, and prednisone (R-CHOP). In a retrospective study of patients with gastrointestinal follicular lymphoma, 89% of whom had D-FL, this regimen resulted in a 93% progression-free survival rate and a 100% five-year overall survival rate [13].

Some studies have reported D-FL regression following treatment of *H. pylori.* For example, in a study by Hayashi et al. a woman with D-FL was treated with antibiotics for *H. pylori.* At the five-year follow-up, there was a complete regression of her D-FL [14]. Another patient with D-FL who received clarithromycin monotherapy reported D-FL regression at a two-year follow-up [15]. These cases illustrate the potential relationship between the eradication of *H. pylori* and regression of D-FL in individuals who present with both simultaneously. Further investigation into the effect of antimicrobials on the regression of D-FL is warranted.

The typical appearance and location of duodenal-type follicular lymphoma should raise awareness of the need to broaden the differential for duodenal polyposis. Although asymptomatic polyps encountered in the duodenum are often considered benign, our case highlights the possibility of malignant potential. Endoscopists should be aware of the typical appearance and location of D-FL, and if encountered, they should rule out extra-duodenal involvement. This is important as it would help with initial staging and present the patient with appropriate treatment options. Our case also reiterates that localized radiotherapy has excellent outcomes for this newly recognized pathology. As the incidence of D-FL rises, future studies should aim to determine a consensus on treatment approaches, particularly for more advanced stages of the disease.

## Conclusions

Although duodenal-type follicular lymphoma generally carries a good prognosis, physicians must be aware of this cancer due to the increased risk of systemic B-cell lymphoma. Endoscopists must also recognize this cancer, as it is typically an incidental finding seen on EGD. This case highlights the role of prompt treatment with localized radiotherapy, resulting in positive outcomes. As our understanding of this pathology evolves, we should better understand its relationship to *H. pylori *eradication and define appropriate therapeutic approaches at advanced stages of disease.
